# Measures of youth e-cigarette use: strengths, weaknesses and recommendations

**DOI:** 10.3389/fpubh.2024.1412406

**Published:** 2024-07-02

**Authors:** Arielle Selya, Martino Ruggieri, Riccardo Polosa

**Affiliations:** ^1^PinneyAssociates, Inc., Pittsburgh, PA, United States; ^2^Center of Excellence for the Acceleration of Harm Reduction (CoEHAR), University of Catania, Catania, Italy; ^3^Unit of Pediatric Clinic, AOU “Policlinico”, PO “G. Rodolico”, Department of Clinical & Experimental Medicine, University of Catania, Catania, Italy; ^4^Department of Clinical & Experimental Medicine, University of Catania, Catania, Italy

**Keywords:** adolescents, behavior, e-cigarettes, electronic nicotine delivery systems, nicotine use, surveillance

## Abstract

This perspective discusses how to best define “e-cigarette use” among youth in a way that is relevant to individual and human health. Commonly-used definitions of youth e-cigarette use have been adapted from measures validated for tobacco cigarette smoking among adults, but may not carry the same meaning for a different product (with a much lower risk profile and very different patterns of use) and a different population (whose use is more often transient and experimental, rather than frequent and persistent). We discuss strengths and weaknesses of different definitions, and recommend improvements in defining youth e-cigarette use. We find that current literature employs a range of definitions of e-cigarette use, from lifetime use (“even a puff”) to daily use. More lenient measures capture more potentially at-risk youth, but much of this is transient experimentation that has negligible risks in and itself, if not persistent. More stringent measures such as daily use are more relevant to individual and public health. Future research should examine possible improvements to definitions which include intensity of use (e.g., number of puffs per day) and persistence/duration of use, either via self-report or technology-assisted data capture.

## 1 Introduction

E-cigarettes are a lower-risk nicotine product that can benefit adults who smoke and are unlikely to quit entirely (1–4), but there are ongoing concerns about youth e-cigarette use. Continued surveillance of youth e-cigarette use is needed, especially considering that use patterns continue to change with the evolving product market. For example, e-cigarettes were introduced into the US market in 2007, but adult current use prevalence remained low (<2%) through at least 2012 (5), after which it fluctuated through 2018 at approximately 3–4% (6). Retail data broadly corroborate these trends, with low sales prior to 2013, and the e-cigarette market increased with Blu in 2013, Vuse in 2014, and JUUL in 2017 (7). Since 2017, US retail trends (primarily reflecting purchases by adults, who comprise a greater share of the population see (8)); e.g., have shifted toward high-nicotine content e-cigarettes (9), and the most common brands in 2022 (Vuse, JUUL, Elf Bar, NJOY, and Breeze Smoke) (10) utilize nicotine-salt formulations, which provide higher nicotine delivery (11). This is beneficial for adult smokers wanting to switch to e-cigarettes but has raised concerns about these products’ addictiveness for youth.

Over this time frame, e-cigarettes have become the most common nicotine product among US youth (12–15) as cigarette smoking reached historic lows (16–20). Youth prevalence of any e-cigarette use in the past 30 days (P30D) peaked in 2019 in the US; this was primarily of JUUL (21, 22). Out of concern over this unacceptably high rate of youth use, Juul Labs, Inc. voluntary discontinued its non-tobacco, non-menthol-flavored products, followed shortly by the U.S. Food and Drug Administration’s (FDA’s) announcement to prioritize enforcement against non-tobacco, non-menthol-flavored pod/cartridge e-cigarettes (23). Subsequently, youth e-cigarette use shifted to sweet- and fruit-flavored disposable products (21) such as Puff Bar in 2021 and 2022 (24, 25) and more recently, Elf Bar (13). Fortunately, youth P30D use has also fallen substantially has fallen by >60% in 2023, compared to its 2019 peak (22), and correspondingly, youth use of JUUL as usual brand fell from 16.3% of all high school students and 5.7% of all middle school students in 2019^1^ (22) to <0.3% of all youth in 2023^2^ (13).

Given that cigarettes are at the most harmful end of the continuum of risk (1–4) and evidence that the two products are substitutes (21, 26–28), it is also important to monitor youth cigarette smoking as e-cigarette us trends change. A related concern is dual use, especially with cigarettes, given the possibility of combined exposures to multiple products. However, reassuringly, accompanying the peak-and-decline in P30D youth e-cigarette use, youth P30D cigarette smoking fell to the all-time low of 1.5% (22). Similarly, P30D use of 2+ products has declined along with overall P30D e-cigarette use, among both high school (from 10.2% in 2020 to 3.9% in 2023) and middle school (from 4.0% in 2020 to 2.5% in 2023) students (13, 29). Several other countries also show a concomitant rise in e-cigarette use and a rapid decline in smoking, including Canada, England, New Zealand, and Germany (30–33). Nevertheless, ongoing surveillance of youth nicotine use is warranted, especially for e-cigarettes, as the most commonly-used product currently.

A necessary element of youth surveillance, as well as comparability of research, is defining “e-cigarette use” consistently across studies and using a measure that has external validity (i.e., relevance to public and individual health). There is currently no clear consensus on how best to define “use,” and the research field would benefit from explicitly weighing different definitions. Here we discuss trends in different current definitions of “e-cigarette use” and corresponding strengths and weaknesses, and make recommendations.

### 1.1 Historical context

Commonly-used metrics for measuring e-cigarette use in both youth and adults seem to have been adapted from those used for *cigarette* smoking in adults, which have been validated against both biochemical markers of exposure (e.g., cotinine or carbon monoxide) and clinical health outcomes. Self-reported measures of smoking – especially measures of daily consumption such as cigarettes per day (CPD) – are generally strongly correlated with biochemical markers of exposure (e.g., cotinine or carbon monoxide) in adults (34, 35), which in turn are associated with adverse health outcomes (36–38). Importantly, however, the concordance between self-reported smoking and exposure levels varies widely across studies, and partly depends on how smoking status is defined (34). Specifically, many light and occasional smokers (e.g., <10 CPD) have similar exposure levels to tobacco-naïve individuals (35, 39), prompting recommendations to define positive smoking status using daily-consumption criteria (e.g., 10+ CPD) to prevent misclassification that could obscure the true impact of regular smoking on health (39).

On the other hand, *duration* of smoking and/or cumulative exposure (e.g., pack-years) are more strongly associated with *clinical* outcomes in adults (e.g., lung cancer, coronary artery disease, and severity of chronic obstructive pulmonary disease) than is CPD alone (40). In fact, one study concluded that “*smoking at a lower intensity for longer duration is more deleterious than smoking at a higher intensity for a shorter duration*” (41).

These validated measures of adult cigarette smoking have been adapted in two separate ways without rigorously evaluating whether these adaptations alter their utility: first, to a different product (from cigarettes to e-cigarettes), and second, to a different population (from adults to youth). Regarding the first adaptation – from cigarettes to e-cigarettes – complications may arise from the fact that e-cigarettes have a much lower risk profile than cigarettes (2), which seems to indicate a higher-threshold definition is warranted to measure an equivalent level of health risk. Additionally, e-cigarettes and cigarettes involve different patterns of use (see below), and thus a given definition of use may be incomparable between the products. Additionally, despite the recommendations from the adult smoking literature to measure quantity and/or duration of cigarette smoking (39–41), not all nationally-representative US surveys collect such information for e-cigarette use (42, 43), limiting the available measures to only current use and resulting in a more lenient definition.

Regarding the adaptation from adults to youth, there are additional complications stemming from the fact that youth use is not typically as heavy or prolonged as adult use, and is more often transient and experimental. For example, smoking is likely to be underreported by underage youth – especially when they have privacy concerns when providing survey responses (44) – which may explain why self-reported nonsmoking individuals can have above-threshold exposure levels (39, 45). Another explanation for this type of discrepancy, suggested by a Statistics Canada publication, is that “*smoking initiation or experimentation in this period may have resulted in some cases being inappropriately classified… particularly among respondents aged 12 to 19*” (35) – the implication being that mere initiation or experimentation should *not* be considered as true smoking. Additionally, there are notable exposure differences in *how* one smokes; youth who did not inhale into their lungs more often had below-threshold exposure levels (45).

Despite the importance of accounting for intensity and/or duration when defining “use,” youth use is often measured using more loosely, defining “current smoking” as *any* smoking (even a puff) in P30D. This low threshold is likely motivated by the fact that “no amount of smoking is safe” (46), and youth cigarette smoking – even low amounts – can be associated with nicotine dependence (47, 48) and potentially lead to long-tern use (49, 50). While little to date is known about how often infrequent *e-cigarette* use leads to long-term chronic use, it could plausibly be expected to be less likely for e-cigarettes than cigarettes, considering that dependence on e-cigarettes is lower than on cigarettes (51, 52). Relatedly, youth measures of smoking are typically less stringent than typical measures of *adult* use, in that youth do not (yet) meet the criteria for “established” use (i.e., cumulative 100 cigarettes/lifetime) or daily consumption (e.g., 10+ CPD) typically used among adults (53), since youth have had less time to accrue this level of use. Thus, adopting a “lower bar/threshold” for measuring youth tobacco cigarette use is often considered appropriate.

While surveillance of cigarette and e-cigarette use is often presented equivalently between youth and adults as “current use” (13, 53), the specific questions are different: adult current use is standardly assessed as use on “some days” or “every day” (vs. “not at all”) (42, 43, 54, 55) while youth current use is standardly assessed as “any use, even a puff, in the past 30 days (P30D)” (13, 15, 56). The two measures are largely consistent with each other, but there is some notable discrepancy: for example, a comparison of the two metrics in young adults found that the standard youth definition yields higher prevalence estimates than the standard adult definition (34.4% vs. 27.3% for “any use in P30D” vs. “some day or every day use,” respectively) (57).

In summary, measures developed and validated for adult cigarette smoking have been adapted in two ways – from cigarettes to e-cigarettes, and from adults to youth – both of which introduce separate sets of complications. These adaptations raise the question of whether these measures remain valid, and call for re-evaluation and, if necessary, improvement of standard metrics for e-cigarette use that are relevant to individual and public health.

### 1.2 Metrics for measuring youth e-cigarette use

Table 1 presents the common definitions of e-cigarette use, which range from lifetime use (i.e., ever had even a single puff) to daily use. While there is no clear consensus in the literature, the most standard measures in the literature are lifetime use, past-12-month (P12M) use, and P30D use, which are used in several US national youth surveys. Also fairly common are frequency-based measures such as use on 20+ days out of P30D and daily use. The exact measure used is important as it can lead to different interpretations; for example, King cites NYTS data, switching between percentages (“*in 2019, current (past-30-day) e-cigarette prevalence reached a peak among middle-school (10.5%) and high-school (27.5%) students*”) and raw numbers (“*nonetheless, in 2021, more than 2 million US middle- and high-school students used e-cigarettes*”) (63), which obscures the magnitude of the decline after 2019.

**Table 1:** Common definitions of youth e-cigarette use.

Definitions	Explanation	Examples of surveys and publications	Estimated relevance to human health, and rationale
Lifetime use / ever-use	Ever using an e-cigarette once, even a single puff	Surveys: MTF, NYTS, PATH, YRBS Studies: (13, 24)	Negligible absolute risk; majority of ever-use does not persist even to P30D use (13, 58), let alone to long-term durations that, for more-harmful cigarette smoking, are linked to health outcomes (40, 41).
Past-12-month (P12M) use	Using an e-cigarette at least once in the past 12 months	Surveys: PATH Studies: (59)	Negligible absolute risk; P12M *cigarette* smoking is rarely followed by continued and established use a year later (59), and this may be less likely for e-cigarettes as they are associated with lower dependence (51, 52).
Past-30-day (P30D) use	Using an e-cigarette at least once in the past 30 days	Surveys: MTF, NYTS, PATH, YRBS Studies: (13, 24, 60)	Probably no absolute risk for less frequent (e.g., 1 day in P30D) and less intense (e.g., 1 puff/day) use patterns, as even for more harmful cigarettes, biomarkers of exposure for <10CPD are often indistinguishable from nonsmoking (35, 39); additionally, most P30D use is very infrequent (1–5 days in P30D) (13), indicative of experimentation that is often transient rather than persistent. However, risk increases with more frequent & intense use (35, 39), and with longer durations of use (40, 41).
Frequent use	Using e-cigarettes on 20+ days out of P30D	Surveys: NYTS, PATH Studies: (13, 24)	May pose some risk; but less risk for very light use (e.g., 1 puff/day), as even for more harmful cigarettes, biomarkers of exposure for <10CPD are often indistinguishable from nonsmoking (35, 39). However, risk increases with more frequent & intense use (35, 39), and with longer durations of use (40, 41).
Near-daily use	Using e-cigarettes on 25+ days out of P30D; often sometimes misleadingly referred to as “daily use”	Surveys: TLC Studies: (61)	Likely poses some risk; risk depends on intensity of use (e.g., # puffs per day), just as with more-harmful cigarettes (e.g., <10CPD often produces similar exposure levels as nonsmoking) (35, 39). Risk also depends on how long near-daily use persists (40, 41), just as with more-harmful cigarettes (e.g., quitting before age 40 avoids most of the premature mortality) (62).
Daily use	Using e-cigarettes on every day in P30D	Surveys: MTF, NYTS, PATH Studies: (13, 24)	Likely poses some risk; risk depends on intensity of use (e.g., # puffs per day), just as with more-harmful cigarettes (e.g., <10CPD often produces similar exposure levels as nonsmoking) (35, 39). Risk also depends on how long near-daily use persists (40, 41), just as with more-harmful cigarettes (e.g., quitting before age 40 avoids most of the premature mortality) (62).

MTF, Monitoring the Future; NYTS, National Youth Tobacco Survey; PATH, Population Assessment of Tobacco and Health; TLC, Truth Longitudinal Cohort; YRBS, Youth Risk Behavior Survey.

## 2 Discussion

### 2.1 Strengths and weaknesses of existing measures of use

Broadly, the main distinction between the common definitions presented in Table 1 is the frequency of use. Note that these measures do not include information on daily consumption/*intensity* of use (e.g., # puffs per day), which is often not captured at all in surveys.

On one hand, lenient definitions (e.g., lifetime use, P12M use) have both conceptual and practical advantages: as noted above for cigarette smoking, the first use of an e-cigarette is not harmful *in and of itself*, but *could* lead to long-term and problematic use (49, 50), which could motivate capturing all youth *potentially* at risk. Practically, lenient definitions capture greater numbers of youth, making statistical analyses easier, as opposed to more stringent definitions yielding too few youth to statistically analyze (Table 2), even in large nationally-representative surveys (59).

The drawback of using lenient definitions of use is that they capture a large fraction of experimental use that does not evolve into long-term use and (if not) poses negligible harms to human health. For example, data from NYTS 2022 and 2023 show that less than half of the youths who *ever* used e-cigarettes *persisted* in using them in the P30D (13, 58). P30D use also includes some level of experimental use, especially if one-time experimentation occurs in the month preceding the survey. Among youths who reported using e-cigarettes in the P30D in NYTS 2023, more (46.1%) used e-cigarettes on only 1–5 days in the P30D period than used them *frequently* (i.e., on 20+ days out of the P30D period; 34.7%) (13). Few used on intermediate number of days (19.1% used on 6–19 days), confirming the bimodal frequency distribution observed for nicotine product use (64). This suggests that *near-daily* (sometimes misleadingly referred to as “daily” use (61)) or *daily* P30D use, rather than *any* P30D use, is more relevant to health risks.

Additionally, *any* P30D use often does not lead to continued/persistent use over time. For example, an analysis of product-use transitions in PATH study showed that approximately one-quarter of youths who exclusively used e-cigarettes in the P30D were not using either e-cigarettes or cigarettes the following year (65). In a more recent study of youth and young adults (ages 15–24) in Ohio, US, very infrequent use (i.e., on ≤5 days in P30D) was found to be highly stable over time, with 76.8% maintaining the same behavior 12 months later (66). In fact, using on ≤5 days in P30D was at least as stable as more frequent use (i.e., on 6+ days in P30D): the probability of maintaining ≤5 vs. 6+ use days over 4 months was 81.5% vs. 73.1%, though the significance of this difference was not tested (66). Definitions that include information on *persistence* or continued use were proposed by Sun et al. (59) in the context of *cigarette* smoking (Table 2), and could reasonably be extended to e-cigarette use. The first definition is rather lenient, capturing initiation in the P12M, and subsequent definitions are increasingly strict. The number of youth captured by each additional criterion drops rapidly; even adding one additional lenient criterion of P12M use 1 year later drops the number of youth meeting criteria for “use” by ~40%. Arguably, the most stringent definition (use at multiple timepoints, leading to established and lifetime use) is the most indicative of problematic patterns of use; however, its prevalence is vanishingly small, comprising only 3% of youth captured by the most lenient definition, and is too few to statistically analyze (59).

**Table 2:** Examples of increasingly stringent measures of use, from Sun et al. (59).

Definition of Cigarette Smoking	# of participants (of 8,671 total)	Weighted % of population
Initiated P12M smoking between Waves 3 and 4	362	4.1%
P12M use at Wave 4 and P12M use at Wave 5	218	2.5%
P12M use at Wave 4 and P30D use at Wave 5	133	1.5%
P12M use at Wave 4 and established use at Wave 5	60	0.8%
P12M use at Wave 4, established use, and use on ≥5 days in P30D at Wave 5	27	0.4%
P12M use at Wave 4, established use, and use on ≥20 days in P30D at Wave 5	12	0.2%

Analysis is based on PATH Waves 3–5. Established use: 100 + cigarettes/lifetime. P12M: past 12-months. P30D: Past 30 days. Source: Sun et al. (59).

Overall, more stringent measures such as daily and persistent use better isolate truly problematic use patterns. The concerns about long-term health effects and nicotine dependence (24, 67) are moot if initial experimentation does not evolve into regular, long-term use. Even for tobacco cigarettes – which pose significantly greater risks than e-cigarettes (1, 2) – stopping smoking before the age of 40 has been shown to substantially reduce risks of dying from smoking-related diseases (62).

### 2.2 Recommendations for future research

Ongoing research is needed on which measures of youth e-cigarette use may best distinguish transient, experimental use from truly problematic patterns of use (i.e., high daily consumption, frequent use, and/or long duration of use). Table 2 shows the importance of assessing continuous e-cigarette use over long time periods; however, many youth surveys are cross-sectional in nature, and cannot prospectively assess persistent use. Future research could examine the accuracy of retrospective self-reported duration or persistent use. Additionally, alternative definitions could include measures of e-cigarette daily use *intensity,* such as number of puffs or puffing sessions per day. Xie et al. recently validated number of puffs per month against cravings and low intention to quit (68); future research is needed to further validate against dependence scales and subsequent use patterns (especially frequent and persistent use), and on how to most accurately collect intensity data (e.g., self-reports vs. data collected with digital tracking tools).

Another consideration is that use patterns differ between cigarettes and e-cigarettes, which may impact the relevance of different measures of use. For example, a “use occasion” for a cigarette is typically finishing an entire cigarette, but e-cigarettes are often consumed in smaller amounts but more frequently – a pattern that has been described as “grazing” (69, 70). These different use patterns, along with the above inability to consistently distinguish exposure levels of low-level smoking vs. non-use, demonstrate that measures of use should not be assumed to be equivalent across products. Similarly, dependence measures cannot be assumed to be equivalent across products, and in fact in some cases are shown to be incomparable (71).

### 2.3 Limitations

There are many considerations, sometimes conflicting, in how to best assess “use.” For example, validation against biochemical exposures vs. clinical outcomes identifies different self-report variables as important (CPD vs. duration, respectively). Measures of use are probabilistic and imperfect: even daily use (which we identify as likely relevant to health outcomes) will capture some youth who will not persist to established, long-term use; and will miss others who do at a later point in time. Further complications arise from standardizing measures of use across the diversity of e-cigarette products, such as differences in nicotine delivery and possible harmful exposures due to product characteristics (e.g., freebase nicotine vs. nicotine salts, different nicotine concentration, device power, and flavors). Much remains unknown about the validity of different definitions, and pros and cons must be weighed – which we aim here to elucidate.

## 3 Conclusion

It is regrettable that the metrics currently employed to evaluate youth e-cigarette usage have been directly borrowed from those used for *cigarette* smoking in adults, without re-evaluating whether their validity holds for a different product (with different use patterns and a much lower risk profile) and in a different population (whose use patterns are more often transient and experimental). Definitions of use that are more indicative of truly problematic measures of use must include criteria for continuous use over some time, cumulative lifetime use, and frequent use. Methods offering objective and precise data collection about the intensity of e-cigarette use (e.g., # puffs) like digital tracking tools, mobile applications and sensor technology are likely to be most valuable, though additional validation work is needed. More generally, the wide range of measures currently used has a correspondingly wide range of prevalence estimates; low thresholds have greater “capture” and may evoke emotional responses that are not grounded in quantification of the actual risks to individual and public health. Forthcoming research, therefore, would benefit from providing a “data interpretation guide” that specifies the relevance of each study’s selected measure(s) of use to individual and public health.

## Data availability statement

The original contributions presented in the study are included in the article/supplementary material; further inquiries can be directed to the corresponding author.

## Ethics statement

Ethical approval was not required for the study involving humans in accordance with the local legislation and institutional requirements. Written informed consent to participate in this study was not required from the participants or the participants’ legal guardians/next of kin in accordance with the national legislation and the institutional requirements.

## Author contributions

AS: Writing – review & editing, Writing – original draft, Project administration, Investigation, Conceptualization. MR: Writing – review & editing. RP: Writing – review & editing, Supervision, Investigation, Conceptualization.

## Footnotes

^1^Estimated as: 27.5% of high school students who used e-cigarettes in P30D × 59.1% of P30D users who listed JUUL as usual brand; and 10.5% of middle school students who used e-cigarettes in P30D × 54.1% who listed JUUL as usual brand (22).

^2^Estimated as 7.7% of all youth who used e-cigarettes in P30D × 3.4% of P30D users who listed JUUL as usual brand (13).
